# Neuronal deletion of Ca_V_1.2 is associated with sex-specific behavioral phenotypes in mice

**DOI:** 10.1038/s41598-022-26504-4

**Published:** 2022-12-22

**Authors:** Annette J. Klomp, Ashley Plumb, Jacqueline B. Mehr, Deniz A. Madencioglu, Hsiang Wen, Aislinn J. Williams

**Affiliations:** 1 Iowa Neuroscience Institute, University of Iowa, Iowa City, IA USA; 2 Department of Psychiatry, University of Iowa, Iowa City, IA USA; 3 Department of Physical Therapy and Rehabilitation Science, University of Iowa, Iowa City, IA USA; 4 Department of Psychiatry, Robert Wood Johnson Medical School, Rutgers University, Piscataway, NJ USA; 5 Brain Health Institute, Rutgers University, Piscataway, NJ USA

**Keywords:** Ion channels in the nervous system, Learning and memory, Motor control, Social behaviour, Autism spectrum disorders

## Abstract

The gene *CACNA1C*, which encodes the pore forming subunit of the L-type calcium channel Ca_V_1.2, is associated with increased risk for neuropsychiatric disorders including schizophrenia, autism spectrum disorder, major depression, and bipolar disorder. Previous rodent work identified that loss or reduction of Ca_V_1.2 results in cognitive, affective, and motor deficits. Most previous work has either included non-neuronal cell populations (haploinsufficient and Nestin-Cre) or investigated a discrete neuronal cell population (e.g. CaMKII-Cre, Drd1-Cre), but few studies have examined the effects of more broad neuron-specific deletion of Ca_V_1.2. Additionally, most of these studies did not evaluate for sex-specific effects or used only male animals. Here, we sought to clarify whether there are sex-specific behavioral consequences of neuron-specific deletion of Ca_V_1.2 (neuronal Ca_V_1.2 cKO) using Syn1-Cre-mediated conditional deletion. We found that neuronal Ca_V_1.2 cKO mice have normal baseline locomotor function but female cKO mice display impaired motor performance learning. Male neuronal Ca_V_1.2 cKO display impaired startle response with intact pre-pulse inhibition. Male neuronal Ca_V_1.2 cKO mice did not display normal social preference, whereas female neuronal Ca_V_1.2 cKO mice did. Neuronal Ca_V_1.2 cKO mice displayed impaired associative learning in both sexes, as well as normal anxiety-like behavior and hedonic capacity. We conclude that deletion of neuronal Ca_V_1.2 alters motor performance, acoustic startle reflex, and social behaviors in a sex-specific manner, while associative learning deficits generalize across sexes. Our data provide evidence for both sex-specific and sex-independent phenotypes related to neuronal expression of Ca_V_1.2.

## Introduction

L-type voltage gated Ca^2+^ channels (LVGCCs) are transmembrane proteins that allow influx of Ca^2+^ into excitable cells. Of the four LVGCCs, Ca_V_1.2 is the predominant isoform in the mammalian brain. Ca_V_1.2 has roles in dendritic development, synaptic plasticity, neuronal survival, and cognitive and affective behaviors^1–4^. The alpha 1C subunit of Ca_V_1.2 is encoded by the gene *CACNA1C*, which has been robustly linked to increased risk for many neuropsychiatric disorders including autism, schizophrenia, major depression, and bipolar disorder^5–11^. Ca_V_1.2 haploinsufficient mice, which lack one copy of Ca_V_1.2 in all tissues, exhibit antidepressant-like phenotypes, hypoactivity, anxiety-like behaviors, increased fear responses, and changes in social behavior^11–17^. However, these mice have reduced Ca_V_1.2 expression in all tissues throughout the body; therefore, it is unclear from Ca_V_1.2 haploinsufficient mice which cognitive, motor, and affective behaviors are directly related to Ca_V_1.2 expression in neurons.

Interestingly, epidemiological data shows that some disorders associated with genetic variation in Ca_V_1.2, such as autism, attention deficit hyperactivity disorder, and major depression, have disproportionate representation in humans of different sexes. Lifetime prevalence of major depressive disorder is higher in females compared to males across the globe^18,19^. In the US, females are more likely to have affective or anxiety disorders than males^20^. In contrast, attention deficit hyperactivity disorder is more prevalent in males than in females in Canada^21^ and the US^22^, and autism is more common in males than females in the US^23,24^. Furthermore, the presentation of these disorders can vary greatly by sex. For example, autistic females tend to present with fewer restricted and repetitive behaviors, fewer externalizing behaviors, and lower nonverbal IQ^24–26^. These epidemiological data raise the possibility that alterations in Ca_V_1.2 are associated with sex-specific phenotypes.

There is some evidence for sex-specific phenotypes due to Ca_V_1.2 deficiency in rodent models. Ca_V_1.2 haploinsufficient female mice were found to have decreased acoustic startle response, decreased learned helplessness, decreased risk-taking behavior and increased anxiety relative to control females^11^. In male Ca_V_1.2 haploinsufficient rats, emission of pro-social 50-kHz ultra-sonic vocalizations during rough-and-tumble play was reduced compared to their control littermates, but play behavior was not affected. Female Ca_V_1.2 haploinsufficient rats displayed more social play behavior and higher levels of pinning behavior than female control littermates^16,27^. However, no sex differences were noted in neuron-specific Ca_V_1.2 conditional knockout mice in anxiety, spatial learning, or fear conditioning tasks when the conditional deletion was driven by Synapsin1-Cre (Syn1-Cre)^28,29^. These data suggest that some brain circuits may be altered by loss of Ca_V_1.2 in a sex-specific manner.

We explored whether neuron-specific deletion of Ca_V_1.2 generates sex-specific phenotypes in cognitive, affective, motor, and social behaviors. To delete Ca_V_1.2 from neurons we used the Syn1-Cre driver line, which targets most central nervous system neurons but spares glia and most cerebellar neurons^30,31^, and results in the loss of the majority of Ca_V_1.2 protein from brain tissue^31^. Previous work in this conditional knockout model where Ca_V_1.2 deletion is driven by Syn1-Cre (neuronal Ca_V_1.2 cKO) identified a significant deficit in extinction of fear learning^28,29^ and a shift in inhibitory/excitatory balance in the lateral amygdala^28^, without abnormalities in locomotor function, spatial memory, or anxiety^28,29^. Intriguingly, we observed both sex-specific and sex-independent behavioral phenotypes in neuronal Ca_V_1.2 cKO mice. Female Ca_V_1.2 cKO mice displayed impaired motor performance learning on the rotarod and decreased social interaction time on the three-chamber social preference task, while male Ca_V_1.2 cKO mice exhibited decreased social preference and acoustic startle response. Both male and female Ca_V_1.2 cKO mice displayed impaired associative learning and altered gait duration on the Erasmus Ladder and increased latency to float on the forced swim test. No sex or genotype differences were noted in baseline locomotor function, sensorimotor gating, or cerebellar-dependent associative learning. Neuronal Ca_V_1.2 cKO mice also showed no behavioral phenotypes in the elevated zero maze, thigmotaxis in the open field, or sucrose preference. Our results suggest that loss of neuronal Ca_V_1.2 is associated with sex-specific phenotypes in motor, social and acoustic startle behaviors, while cognitive phenotypes generalize across sexes.

## Methods

### Mice

The generation of neuron-specific Ca_V_1.2 conditional knockout (cKO) mice has been described previously^29,31^. Briefly, mice with a floxed Ca_V_1.2 exon 2 allele (Ca_V_1.2^f/+^ or Ca_V_1.2^f/f^, maintained on a 129SvEv genetic background)^32^ were first bred to transgenic mice expressing the Cre recombinase driven by the Synapsin1 promoter (Syn1-Cre^Cre/+^, maintained on a C57BL/6N background)^30^, producing an F1 cross. Using non-littermate offspring from the F1 generation, Ca_V_1.2^f/+^ Syn1-Cre^Cre/+^ mice were then crossed with Ca_V_1.2^f/+^ Syn1-Cre^+/+^ mice to produce Ca_V_1.2^f/f^ Syn1-Cre^Cre/+^ conditional knock-out (cKO) mice as well as control littermate (CTRL) mice. No significant differences have previously been detected between Ca_V_1.2^+/+^ Syn1-Cre^+/+^, Ca_V_1.2^f/f^ Syn1-Cre^+/+^, Ca_V_1.2^f/+^ Syn1-Cre^+/+^, and Ca_V_1.2^+/+^ Syn1-Cre^Cre/+^ mice^28,29^; therefore these groups were collapsed into a single CTRL group. Breeding pairs always consisted of a Cre positive female (Cre/+) and a Cre negative male (+/+) to avoid germline recombination in offspring as described previously in the Syn1-Cre mouse line^33^. All mice were adults (10–25 weeks old) at the time of testing. Cohort 1 completed the following tasks in this order: elevated zero maze, open field test, three chamber social preference test, rotarod, forced swim test, and sucrose preference test. Cohort 2 was tested on Erasmus Ladder. Cohort 3 underwent prepulse inhibition. Experimenters were blinded to genotype throughout behavioral testing. Sample sizes are indicated in each figure. For fluorescent assays of Syn1-Cre deletion, L10-eGFP mice^34^ (JAX #024750, gift of Dr. Joel Geerling) were crossed to Syn1-Cre mice to generate Syn1-GFP mice. All experiments were conducted according to the National Institute of Health guidelines for animal care and were approved by the Institutional Animal Care and Use Committee at University of Iowa (Protocol Numbers 7082044 and 0082044). Study details are in accordance with ARRIVE guidelines.

### Histology and microscopy

Syn1-GFP mice were anesthetized at 4 weeks of age with 17.5 mg/ml Ketamine/2.5 mg/ml Xylazine at a dose of 0.1 ml per 20 g and perfused with 4% paraformaldehyde in 0.1 M phosphate buffer (PB). Whole brains were dissected and immersed in 30% sucrose for 72 h. Brains were rinsed in PB and frozen in optimal cutting temperature compound. Brain tissue was sectioned on a cryostat into 50-μm-thick sections. Sections were incubated with DAPI Solution (Thermo Scientific) 1:1000 in PB for 1 min and mounted with Prolong Diamond Antifade Mountant (Invitrogen). Sections were imaged at 20× on an Olympus IX83 fluorescence microscope and stitched together using Olympus cellSens Dimension 2.3 software.

### Western blot

Adult mice were euthanized with 17.5 mg/ml Ketamine/2.5 mg/ml Xylazine at a dose of 0.1 ml per 20 g, then brains were rapidly removed. The cerebrum and cerebellum were immediately dissected and snap frozen in liquid nitrogen. Brain tissues were lysed in ice-cold RIPA buffer and equal amounts of protein were resolved on 7.5% Mini-PROTEAN TGX Stain-Free Precast Gels (Bio-Rad, Hercules, CA) then transferred to polyvinylidene difluoride (PVDF) membranes using the Bio-Rad Trans-Blot Turbo™ Transfer System (Hercules, CA). Membranes were blocked for 30 min with 5% nonfat dry milk, and primary antibody was applied for 2 h at room temperature or overnight at 4 °C. Blots were washed three times with Tris-buffered saline, then incubated with peroxidase-conjugated secondary antibody for 1 h at room temperature, followed by three more washes as described above. Blots were first probed with rabbit αCa_V_1.2^31^ at 1:5000 (#ACC-033, Alomone Labs, Jerusalem, Israel) followed by goat anti-rabbit-HRP 1:5000, then mouse αNa^+^/K^+^-ATPase^35^ at 1:100 (A5, Developmental Studies Hybridoma Bank, Iowa City, IA) followed by anti-mouse-HRP 1:8000 (Jackson Immunoresearch, West Grove, PA). Proteins were visualized with ECL SuperSignal™ West Dura Extended Duration Substrate (Thermo Fisher Scientific 34075), according to the manufacturer’s protocol; signals were captured using a LI-COR Odyssey Fc Imager running Image Studio 5.2.5 (Lincoln, NE). Blots were not stripped between probing for Ca_V_1.2 and Na^+^/K^+^-ATPase.

### Behavioral procedures

#### General

Mice were housed under regular light cycle with lights on/off at 0900/2100 DST (0800/2000 non-DST). The average ambient temperature was 22 °C. Mice were provided with food and water ad libitum, and enriched paper bedding was used in all home cages in a specific pathogen free housing facility. Experimenters were gowned, capped, and gloved at all times. All experiments were conducted during the animals' light cycle. All mice were group housed and estrus cycles were not synchronized nor tracked. All equipment was cleaned between trials with 70% ethanol.

#### Open field

Mice were placed in a 40 cm × 40 cm arena for 10 min under ~ 115–130 lx. Activity was tracked by EthoVision 14 software (Noldus, Leesburg, VA) and analyzed for total distance traveled and thigmotaxis (the tendency to stay at edge of the arena). For the latter, the arena was divided into the periphery and the center where each comprised 50% of the total surface area of the arena.

#### Rotarod

Mice were placed on the rotating drum of an accelerating rotarod (IITC Life Science Mouse, Woodland Hills, CA) to assess motor performance learning, and the time to fall or second passive rotation was recorded for each mouse. The speed of the rotarod accelerated from 4 to 40 rpm over a 5-min period. Mice were given 3 trials/day for 5 days with a maximum trial duration of 5 min, with at least a 10-min inter-trial interval. Latency to fall or second passive rotation were recorded for each mouse each day.

#### Erasmus Ladder

Mice were subjected to the Erasmus Ladder task (Noldus, Wageningen, The Netherlands) which has been described in detail elsewhere^36^, with some modifications. Briefly, the mice were trained on the Erasmus Ladder for 42 trials per day for a total of 4 consecutive days to assess motor coordination and gait adaptation (days 1–4). Trials were separated by a random inter-trial interval ranging from 11 to 20 s. Trials initiated with a light cue. If the mice left the chamber before the light cue or did not leave following the light cue, an aversive air puff would start. Cerebellar-dependent associative motor learning was assessed on an additional 4 consecutive days (days 5–8). Mice were conditioned using a 2 kHz (75 dB) tone (conditioned stimulus, CS) to predict an elevated ladder rung (unconditioned stimulus, US) which the mice had to jump over. The CS preceded the US by 250 ms. The US is also referred to as the perturbation.

#### Forced swim test

Mice were placed in clear acrylic cylinders (outer diameter: 23 cm, inner diameter: 21.5 cm, height: 34 cm) filled halfway with water maintained at 20–25 °C, and video recorded for 6 min. Trials were analyzed for latency to float and percentage of time immobile during the last 4 min of the trial.

#### Sucrose preference test

Mice were habituated to single housing with two burettes filled with water for 2 days prior to data collection. On day 1 of testing, one burette was filled with water and the other with 2% sucrose. Twenty-four hours later, burette volumes were recorded and positions were switched to control for any side position preferences in the mice. The burette volumes were measured and positions were switched daily for an additional 2 days (total of 2 days with each burette in each position). Data are presented as the amount of sucrose consumed as a percentage of total liquid consumed over the 4-day testing period.

#### Elevated zero maze

Mice were placed in a custom-built white plastic maze elevated 42.5 cm off the table with an internal diameter of 33.7 cm and outer diameter of 46 cm (internal pathway 5.8 cm wide). Walls on the closed sections were 10 cm high, and the lip on open sections was 0.6 cm high. Each mouse underwent a single 5-min trial under ~ 250 lx (open sections). Activity was tracked by EthoVision 14 software for distance traveled, velocity, and duration spent in open/closed sections.

#### Three-chamber social preference test

Mice were placed in a matte, black plastic rectangular arena (L × W × H = 51 cm × 25.4 cm × 25.4 cm) divided into three compartments, with a 10 cm wide opening between compartments and empty clear acrylic perforated cylinders in the center of each outer compartment. Mice were habituated to the entire testing apparatus for 10 min. For the preference test, a novel conspecific mouse matched for age, sex, and weight (+ /− 5 g) was placed under one cylinder while a novel object (colored plastic blocks) was placed under the other cylinder, and the test mouse was allowed to explore for 10 min. Mice were placed in middle compartment at the beginning of the test. Mice were removed from the apparatus if they climbed to the tops of the walls of the arena. Activity was tracked by EthoVision 14 software for distance traveled, velocity, and interaction time (calculated by measuring the total duration during which the nose-point, but not the center-point, of the animal was within 1.5 cm of cylinder—a method designed by Benice and Raber^37^ to exclude instances where the mouse was rearing or climbing on the cylinder).

#### Prepulse inhibition

This protocol has been described previously^38^. In short, mice were placed in an isolation cabinet (width: 38.1 cm × diameter: 35.6 cm × height: 45.7 cm), restrained in a clear acrylic cylinder (length: 12.7 cm × inner diameter: 3.8 cm), and the tremble response of the animal was measured via an accelerometer underneath the chamber. The testing apparatus consisted of a startle response box (SR-LAB, San Diego Instruments). Mice were habituated to the testing chamber for 10 min with a consistent background white noise level of 65 dB which was present for the entire experiment. Each mouse underwent 64 trials with the first and last 6 trials (Block I and Block III) consisting of solely pulse alone trials to verify that no acclimation to the startle pulse occurred over the course of the experiment. All trials were presented with a randomly spaced inter-trial interval ranging from 7 to 15 s. The startle pulse was set to 120 dB and the prepulse intensities were set to 5, 10, and 15 dB above the background noise in the testing room. The startle response was recorded in millivolts in SR-LAB software and percent prepulse inhibition was calculated normalized to the startle response of the pulse alone trials from Block II as follows: percent prepulse inhibition = (startle response for pulse alone − startle response for pulse with prepulse)/startle response for pulse alone × 100.

### Statistics

All data were analyzed to assess for sex as a biological variable. Data were graphed and analyzed using GraphPad Prism 8.0 (GraphPad Software, San Diego, CA) and R (R 4.1.1, emmeans 1.7.0, lme4 1.1.27.1, lmerTest 3.1-3, effectsize 0.5), and are graphically represented as mean ± standard error of the mean (SEM) for each group. Normality tests were run to determine the appropriate statistical method for analysis of each experiment. Outlier tests were run using ROUT with a Q = 0.1% and animals were removed only if they were called as outliers on more than 50% of days (this resulted in one CTRL mouse being excluded from Erasmus Ladder misstep analysis). Data were analyzed using the statistical test noted in results and figure legends (linear mixed model, two-way repeated measures ANOVA, one-way ANOVA, or Student’s *t*-tests with appropriate follow-up testing). Results were considered significant when *p* < 0.05 (denoted in all graphs as follows: *p < 0.05; **p < 0.01).

### Ethical approval

All experiments were conducted according to the National Institute of Health guidelines for animal care and were approved by the Institutional Animal Care and Use Committees at University of Iowa. All the listed authors approved the delivery of the manuscript for publication.

## Results

### Ca_V_1.2 cKO mice display normal locomotor and exploratory behavior but females exhibit a motor performance deficit

**Figure 1:**
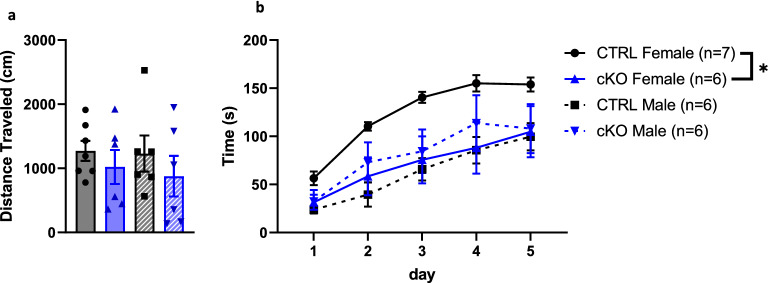
Loss of neuronal Ca_V_1.2 causes impaired motor performance learning in female but not male mice. (**a**) No differences in locomotor activity were detected in distance traveled in the open field test between neuronal Ca_V_1.2 cKO mice and CTRL littermates. (**b**) Female neuronal Ca_V_1.2 cKO mice display a shorter latency to fall than CTRL littermates on the accelerating rotarod. No differences in rotarod performance were detected between CTRL and cKO males. Data are expressed as mean ± s.e.m, using EthoVision 14, www.noldus.com/ethovision-xt.

We first sought to determine whether Ca_V_1.2 cKO mice display abnormal locomotor or exploratory behaviors. In the open field test, we observed no differences in distance traveled by Ca_V_1.2 cKO mice compared to CTRL littermates (CTRL v cKO, 2-way ANOVA, main effect genotype, F_1,21_ = 1.40, *p* = 0.25, main effect sex, F_1,21_ = 0.13, *p* = 0.72, interaction effect, F_1,21_ = 0.04, *p* = 0.84) (Fig. 1a). Given that Ca_v_1.2 has been linked to disorders that involve abnormal motor learning, such as autism and schizophrenia, we hypothesized that neuronal Ca_V_1.2 may be involved in motor performance learning. Therefore, we tested the ability of mice lacking neuronal Ca_V_1.2 to learn the accelerating rotarod task. We observed a genotype × sex interaction (Linear mixed effect model, genotype × sex interaction effect, F_1,21_ = 5.88, *p* < 0.03), and a main effect of day (Linear mixed effect model, main effect day, F_4,84_ = 54.55, p < 0.01), but no main effect of sex or genotype (Linear mixed effect model, main effect genotype, F_1,21_ = 1.20, *p* = 0.29, main effect sex, F_1,21_ = 2.88, *p* = 0.10) (Fig. 1b). All groups demonstrated the ability to learn the accelerating rotarod itself, as they improved between days 1 and 5 (Tukey’s post hoc multiple comparisons test, *p* < 0.01). Follow up testing on the genotype × sex interaction effect showed no differences in motor learning between male CTRL and cKO mice (estimated marginal means, males, t_21_ = − 0.92, *p* = 0.37), but there was a significant difference between female CTRL and cKO mice (estimated marginal means, females, t_21_ = 2.53, *p* < 0.02). Furthermore, there was a difference between CTRL female and male mice (estimated marginal means, CTRL, t_21_ = 2.97, *p* < 0.01) but there was no difference between cKO female and male mice (estimated marginal means, cKO, t_21_ = − 0.50, *p* = 0.62) suggesting that the neuronal deletion of Ca_v_1.2 attenuated the sex differences on this task. Neuronal cKO mice did not differ in weight from CTRL littermates, although we did observe a main effect of sex with female mice being smaller than males on average (two-way ANOVA, main effect of genotype, F_1,21=_0.88, *p* = 0.36, main effect of sex, F_1,21=_ = 33.65, *p* < 0.01, interaction effect, F_1,21=_ = 0.99, *p* = 0.33) (Supplementary Fig. 1). We conclude that loss of neuronal Ca_v_1.2 causes impaired motor performance learning in females but not males.

### Ca_V_1.2 cKO mice display slower gait and no deficit in cerebellar-dependent associative motor learning

**Figure 2:**
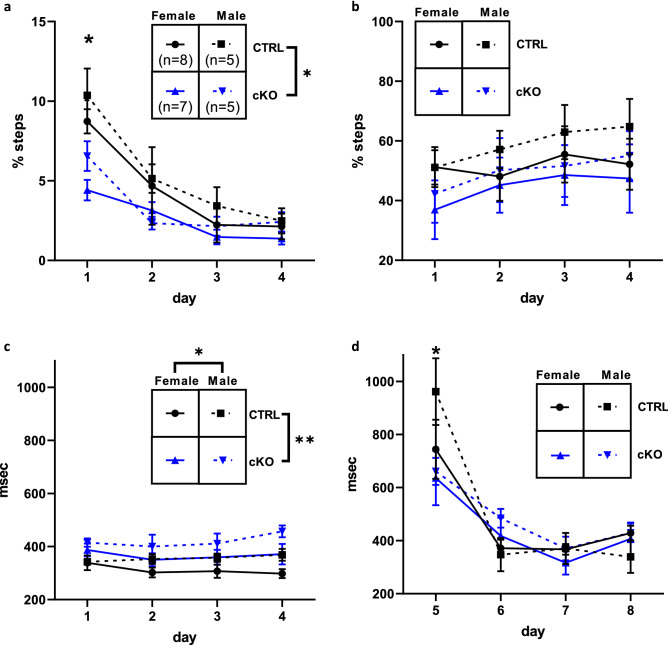
Ca_V_1.2 cKO mice display slower gait but no deficit in cerebellar-dependent associative motor learning on the Erasmus Ladder task. (**a**) Neuronal Ca_V_1.2 cKO mice had fewer missteps than CTRL littermates on day 1 which resolved on days 2–4. (**b**) We observed a main effect of day in the use of long steps, with no sex or genotype-dependent effects. Therefore, on average all groups use more long steps over the course of training, representing normal gait adaptation. (**c**) Neuronal Ca_V_1.2 cKO mice had a longer average step time compared to CTRL littermates. Males also had a longer average step time compared to females. (**d**) All groups learn to jump over a displaced peg in response to a tone cue, shown as a decrease in step time between days 5 and days 6–8. Post hoc testing revealed that CTRL mice had longer post-perturbation step times on day 5 compared to cKO mice, but this was not observed on days 6–8, suggesting that all groups successfully learn to associate the tone with the displaced peg, representative of cerebellum-dependent associative learning. No sex-dependent effects were detected. Data are expressed as mean ± s.e.m, using ErasmusLadder 2.0.214, www.noldus.com/erasmusladder.

We investigated the effect of neuronal deletion of Ca_V_1.2 on gait and cerebellar-dependent learning using the Erasmus Ladder test. On the Erasmus Ladder, mice are assessed on days 1–4 for gait abnormalities and associative learning, then they are tested on days 5–8 for tone-cued cerebellar associative learning. We observed fewer missteps in cKO mice compared to CTRL littermates on day 1, but this resolved on days 2–4 (Days 1–4, Outlier removed, ROUT Q = 0.1%, Linear mixed effect model, genotype × day interaction effect, F_3,60_ = 4.07, *p* < 0.02, main effect genotype, F_1,20_ = 6.90, *p* < 0.02, main effect day, F_3,60_ = 36.06, *p* < 0.01, estimated marginal means, day 1, t = 4.15, *p* < 0.01, estimated marginal means, day 2, t = 1.89, *p* = 0.06, estimated marginal means, day 3, t = 0.99, *p* = 0.33, estimated marginal means, day 4, t = 0.42, *p* = 0.68) (Fig. 2a). As mice learn the Erasmus Ladder task, they transition from using short steps (stepping on each rung) to using long steps (stepping on every other rung), as long steps are a more efficient strategy for crossing the ladder. The transition from short to long steps represents gait adaptation. For short steps, we observed no main effect of genotype or sex and no interaction effect (Days 1–4, Linear mixed effect model, main effect genotype, F_1,21_ = 3.37, *p* = 0.08, main effect sex, F_1,21_ = 2.69, *p* = 0.12) (data not shown). We observed a main effect of day in long steps (Days 1–4, Linear mixed effect model, main effect day, F_3,63_ = 4.92, *p* < 0.01, Tukey’s post hoc multiple comparisons test, day 1 to 3, *p* < 0.01, day 1 to 4, *p* < 0.01) (Fig. 2b) but no main effect of genotype or sex and no interaction effects (Days 1–4, Linear mixed effect model, main effect genotype, F_1,21_ = 0.87, *p* = 0.36, main effect sex, F_1,21_ = 0.50, *p* = 0.49), confirming that all groups display normal gait adaptation during training. We observed a longer average step time in cKO mice compared to CTRL littermates (Linear mixed effect model, main effect genotype, F_1,21_ = 7.50, *p* < 0.02, main effect sex, F_1,21_ = 5.62, *p* < 0.03) (Fig. 2c).

We then measured tone-cued cerebellar dependent associative motor learning using the response to the presentation of an obstacle, a peg raised above the ladder that mice must jump over, immediately following a tone cue on days 5–8. We observed a main effect of day (Linear mixed effect model, main effect day, F_3,63_ = 40.16, *p* < 0.01) and a genotype × day interaction effect (Linear mixed effect model, genotype × day interaction effect, F_3,63_ = 4.93, *p* < 0.01) for the post-perturbation step on days 5–8, showing that all groups learned to jump to avoid the obstacle (Fig. 2d). While post hoc testing revealed that CTRL mice had longer step times on the post-perturbation step on day 5 compared to cKO (estimated marginal means, day 1, t = 2.97, *p* < 0.01), this resolved on days 6–8 (estimated marginal means, day 2, t = − 1.33, *p* = 0.19, estimated marginal means, day 3, t = 0.37, *p* = 0.71, estimated marginal means, day 4, t = − 0.50, *p* = 0.62). Therefore, both CTRL and neuronal Ca_V_1.2 cKO mice demonstrated the ability to perform tone-cued cerebellar-dependent associative motor learning, and neuronal Ca_V_1.2 cKO mice had slightly longer step times than CTRL littermates.

### Ca_V_1.2 cKO mice display deficits in associative learning

**Figure 3:**
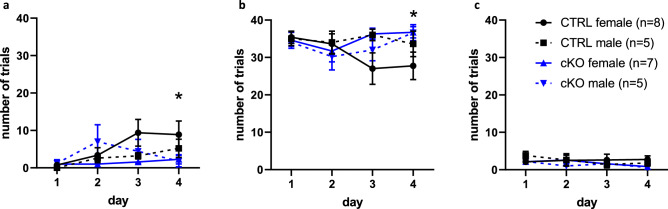
Neuronal Ca_V_1.2 cKO mice have a deficit in associative learning. (**a**) Neuronal Ca_V_1.2 cKO mice do not learn to leave on the light cue whereas CTRL mice do. We found a day × genotype interaction, and post hoc testing showed that cKO mice differed from CTRL mice on day 4. There were no sex-dependent effects. (**b**) Neuronal Ca_V_1.2 cKO mice do not learn to avoid the air cue with trial initiation but CTRL mice do. There was a day × genotype interaction, and post hoc testing showed that cKO mice differed from CTRL mice on day 4. (**c**) There were no differences in how frequently mice walked onto the ladder before any cue was given, suggesting that mice did not display impulsive initiation of the task. Data are expressed as mean ± s.e.m, using ErasmusLadder 2.0.214, www.noldus.com/erasmusladder.

We hypothesized that loss of neuronal Ca_V_1.2 would cause impaired associative learning based on previous studies showing that Ca_V_1.2 is important for certain forms of associative learning. We used the Erasmus Ladder to assess associative learning, which was measured as the ability to learn visual and sensory start cues for each trial. Wild-type mice learned to associate the light cue with the initiation of a trial over the course of training whereas neuronal Ca_V_1.2 cKO mice did not (Linear mixed effect model, genotype × day interaction effect, F_3,63_ = 3.16, *p* < 0.04, main effect day, F_3,63_ = 4.71, *p* < 0.01) (Fig. 3a). Post hoc testing showed that the genotype × day interaction effect was driven by the difference between CTRL and cKO mice on day 4 (estimated marginal means, CTRL v cKO, day 1, t_61_ = − 0.32, *p* = 0.75, day 2, t_61_ = − 0.44, *p* = 0.66, day 3, t_61_ = 1.44, *p* = 0.16, day 4, t_61_ = 2.18, *p* < 0.04). There was no main effect of genotype or sex noted (Linear mixed model, main effect genotype, F_1,21_ = 0.86, *p* = 0.37, main effect sex, F_1,21_ = 0.04, *p* = 0.84). As mice learn the Erasmus Ladder task, they use the air cue less frequently and the light cue more frequently. We observed that CTRL mice learned to use the light cue more frequently over the course of training but cKO mice did not (Linear mixed effect model, genotype × day interaction effect, F_3,63_ = 2.88, *p* < 0.05) (Fig. 3a–b); post hoc testing revealed that CTRL differed from cKO on day 4 (estimated marginal means, CTRL v cKO, day 1, t_61_ = 0.32, *p* = 0.75, day 2, t_61_ = 0.10, *p* = 0.32, day 3, t_61_ = − 0.92, *p* = 0.36, day 4, t_61_ = − 2.09, *p* < 0.05). No main effects of sex or genotype were noted (Linear mixed model, main effect genotype, F_1,21_ = 0.86, *p* = 0.37, main effect sex, F_1,21_ = 0.04, *p* = 0.84). We did not observe any differences between groups in how frequently mice walked onto the ladder before any cue was given (Linear mixed model, main effect genotype, F_1,21_ = 1.15, *p* = 0.30, main effect sex, F_1,21_ = 0.11, *p* = 0.75, main effect session, F_3,63_ = 1.30, *p* = 0.28) (Fig. 3c). These data support the hypothesis that neuronal Ca_V_1.2 cKO mice have a deficit in associative learning.

### Ca_V_1.2 cKO mice display normal sensorimotor gating but males have impaired startle response

**Figure 4:**
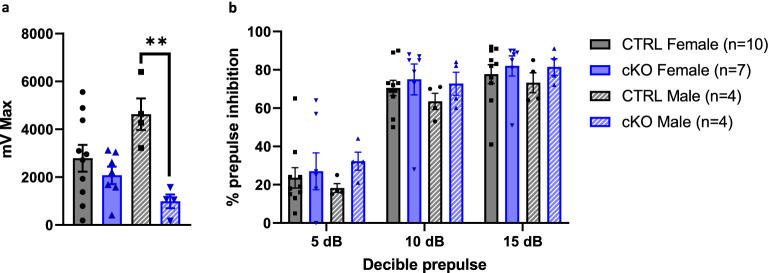
Male neuronal Ca_V_1.2 cKO mice display impaired startle response. (**a**) Male cKO mice have significantly lower acoustic startle response to the auditory startle pulse compared to CTRL littermates. Female cKO mice do not differ from female CTRL mice in acoustic startle response. (**b**) All groups display normal prepulse inhibition of the acoustic startle response, and no sex or genotype differences were observed. Data are expressed as mean ± s.e.m, using SR-Lab, www.sandiegoinstruments.com/product/sr-lab-startle-response.

Given that Ca_V_1.2 has been linked to disorders with impaired sensorimotor gating including autism, schizophrenia, and bipolar disorder, we tested sensorimotor gating and startle response using the prepulse inhibition task. We observed a genotype × sex interaction effect in auditory startle response in neuronal Ca_V_1.2 cKO mice (Linear mixed effect model, main effect genotype, F_1,21_ = 13.07, *p* < 0.01, main effect sex, F_1,21_ = 0.71, *p* = 0.41, genotype × sex interaction effect, F_1,21_ = 7.78, *p* < 0.02); follow up testing showed that male cKO mice have significantly lower response to the auditory startle pulse compared to CTRL littermates (estimated marginal means, female, t_21_ = 0.72, *p* = 0.48, estimated marginal means, male, t_21_ = 3.90, *p* < 0.01) (Fig. 4a). There was a mild habituation to startle response from beginning to end of the experiment for all groups but the startle response was detectable throughout the experiment (Linear mixed effect model, main effect block, F_2,42_ = 3.46, *p* < 0.05) (Supplementary Fig. 2). Interestingly, all groups displayed normal sensorimotor gating as measured by prepulse inhibition (Linear mixed model, main effect genotype, F_1,21_ = 1.72, *p* = 0.20, main effect sex, F_1,21_ = 0.18, *p* = 0.67, main effect type, F_2,42_ = 146.75, *p* < 0.01) (Fig. 4b). Therefore, male neuronal Ca_V_1.2 cKO mice have a specific impairment in startle response, but overall, neuronal Ca_V_1.2 cKO mice have normal sensorimotor gating.

### Male Ca_V_1.2 cKO mice display impaired social preference

**Figure 5:**
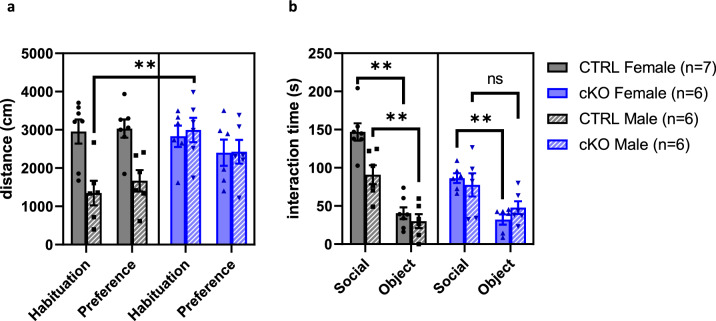
Male Ca_V_1.2 cKO mice display impaired social preference on the three-chamber social preference task. (**a**) Male cKO mice were more active than CTRL littermates during habituation but there were no sex or genotype differences in distance traveled during the preference phase. (**b**) Male Ca_V_1.2 cKO mice do not spend more time with a conspecific mouse compared to a novel object, but all other groups display preference for spending time with a conspecific mouse over a novel object. Female cKO mice also spend less time in social interaction than CTRL female mice. Data are expressed as mean ± s.e.m, using EthoVision 14, www.noldus.com/ethovision-xt.

We tested social preference using the three-chamber social preference test. We first examined distance traveled during the habituation and social preference phases of the task to determine if there were differences between groups in activity during each phase. We observed a genotype by sex interaction and a genotype by phase interaction effect in distance traveled (Linear mixed effect model, main effect genotype, F_1,21_ = 2.20, *p* = 0.15, main effect sex, F_1,21_ = 6.23, *p* < 0.03, main effect phase, F_1,21_ = 1.68, *p* = 0.21, genotype × sex interaction effect, F_1,21_ = 8.06, *p* < 0.01, genotype × phase interaction effect, F_1,21_ = 9.12, *p* < 0.01) (Fig. 5a). Follow up testing showed that male cKO mice were more active during the habituation phase compared to male CTRL littermates (estimated marginal means, CTRL v cKO, habituation male, t_28_ = − 3.80, *p* < 0.01) but there were no differences between female mice during the habituation phase (estimated marginal means, CTRL v cKO, habituation female, t_28_ = 0.30, *p* = 0.77). Furthermore, there were no significant differences between genotypes during the preference phase (estimated marginal means, CTRL v cKO, preference female, t_28_ = 1.51, *p* = 0.14, CTRL v cKO, preference male, t_28_ = − 1.75, *p* = 0.09). Overall, our data show that CTRL males do not traverse this particular arena as much as cKO males either during habituation or social preference testing, but there are no differences in total exploration between CTRL females and cKO females.

When testing for social preference, we observed a main effect of genotype, sex, and cylinder, a genotype by sex interaction, a genotype by cylinder interaction, and a sex by cylinder interaction (Linear mixed effect model, main effect genotype F_1,42_ = 5.10, *p* < 0.03, main effect sex, F_1,42_ = 4.33, *p* < 0.05, main effect cylinder, F_1,42_ = 77.17, *p* < 0.01, genotype × sex interaction effect, F_1,42_ = 6.55, *p* < 0.02, genotype × cylinder interaction effect, F_1,42_ = 8.46, *p* < 0.01, genotype × sex interaction effect, F_1,42_ = 6.55, *p* < 0.02). We found that cKO males do not show a statistically significant preference for conspecific mice over an object (estimated marginal means, social v object, cKO male, t_21_ = − 2.04, *p* = 0.05), which was not attributable to reduced total exploration time in male cKO mice (Fig. 5a). All other groups show a clear social preference (estimated marginal means, social v object, CTRL female, t_21_ = − 7.90, *p* < 0.01, social v object, CTRL male, t_21_ = − 4.17, *p* < 0.01, social v object, cKO female, t_21_ = − 3.73, *p* < 0.01) (Fig. 5b). We also noted that cKO females spent less time interacting with the conspecific mouse compared to CTRL female littermates (estimated marginal means, CTRL v cKO, social female, t_42_ = 4.32, *p* < 0.01) though there were no differences in interaction time with the object (estimated marginal means, CTRL v cKO, object female, t_42_ = 0.60, *p* = 0.55). Furthermore, CTRL females spent more time interacting with the conspecific mouse compared to CTRL males (estimated marginal means, female v male, social CTRL, t_42_ = 3.99, *p* < 0.01). Taken together, these data suggest that male cKO mice have impaired social preference, while female cKO mice display a preference for social interaction over objects but decreased total social interaction time compared to CTRL females. Ca_V_1.2 expression in neurons may differentially affect social behaviors in males versus females.

### Ca_V_1.2 cKO mice display affective-like behaviors that are largely indistinguishable from CTRL littermates

**Figure 6:**
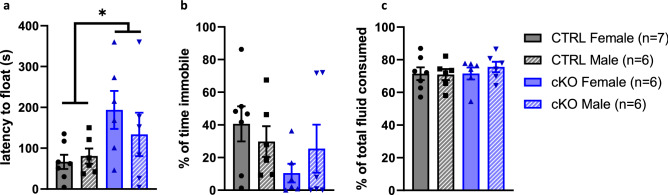
Neuronal Ca_V_1.2 cKO mice exhibit longer latency to float but no other antidepressant-like behaviors. (**a**) Neuronal Ca_V_1.2 cKO mice exhibit a longer latency to float in the forced swim test compared to CTRL littermates. (**b**) Time immobile in the forced swim test did not differ between groups. (**c**) Neuronal Ca_V_1.2 cKO and CTRL mice both prefer sucrose over water. We observed no group differences in sucrose consumption. Data are expressed as mean ± s.e.m.

Since Ca_V_1.2 haploinsufficient mice exhibit antidepressant-like behavior ^13^, we hypothesized that neuronal Ca_V_1.2 cKO would have a similar antidepressant-like phenotype. In the forced swim test, Ca_V_1.2 cKO mice had a significantly longer latency to immobility consistent with an antidepressant-like phenotype (2-way ANOVA, main effect genotype, F_1,21_ = 6.15, *p* < 0.03 main effect sex, F_1,21_ = 0.40, *p* = 0.53, interaction effect, F_1,21_ = 1.05, *p* = 0.32) (Fig. 6a). There appears to be a mild decrease in total time immobile in female cKO mice (Fig. 6b) but this is not statistically significant (2-way ANOVA, main effect genotype, F_1,21_ = 2.60, *p* = 0.12, main effect sex, F_1,21_ = 0.04, *p* = 0.85, interaction effect, F_1,21_ = 1.45, *p* = 0.24). Additionally, no differences were noted in the sucrose consumption of cKO mice (2-way ANOVA, main effect genotype, F_1,21_ = 0.24, *p* = 0.63, main effect sex, F_1,21_ = 0.43, *p* = 0.52, interaction effect, F_1,21_ = 0.41, *p* = 0.53) (Fig. 6c). We conclude that neuronal Ca_V_1.2 cKO mice have increased latency to float but no statistically significant change in total immobility on the forced swim test, and do not display evidence of anhedonia.

### Ca_V_1.2 cKO mice display normal anxiety-like behaviors

**Figure 7:**
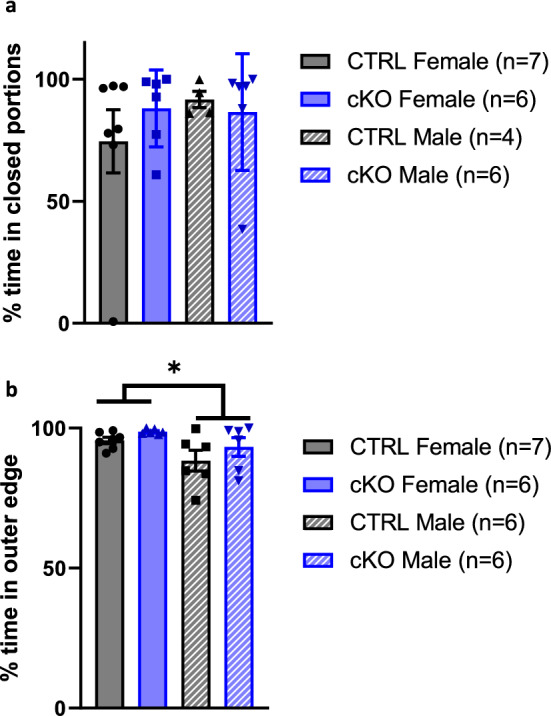
Neuronal Ca_V_1.2 cKO mice display no differences in anxiety-like behaviors. (**a**) There were no sex- or genotype-dependent differences in time spent in the closed portions of the elevated zero maze. (**b**) We observed a sex-dependent difference where female mice spent more time in the periphery of the open field, also called thigmotaxis, compared to male mice. We did not observe any genotype-dependent differences in this task. Data are expressed as mean ± s.e.m, using EthoVision 14, www.noldus.com/ethovision-xt.

We explored anxiety-like behaviors using the elevated zero maze and open field task, which both measure an animal’s tendency to explore a more aversive part of the environment. In the elevated zero maze, we found no differences in percent time spent in the open segments by genotype (2-way ANOVA, main effect genotype, F_1,19_ = 0.16, *p* = 0.69, main effect sex, F_1,21_ = 0.57, *p* = 0.46, interaction effect, F_1,21_ = 0.81, *p* = 0.38) (Fig. 7a). In the open field test, we observed no differences in thigmotaxis by genotype nor was there a genotype × sex interaction effect (Fig. 7b) although we did observe a main effect of sex such that female mice spent more time in the outer edge of the arena compared to male mice (2-way ANOVA, main effect genotype, F_1,21_ = 2.63, *p* = 0.12, main effect sex, F_1,21_ = 6.38, *p* < 0.02, interaction effect, F_1,21_ = 0.14, *p* = 0.72). We conclude that neuronal Ca_V_1.2 cKO mice have no differences in anxiety-like behaviors compared to CTRL littermates.

### Syn1-Cre mediates loss of Ca_V_1.2 expression across most neurons of most regions in the brain

We investigated the expression pattern of Syn1-Cre by crossing Syn1-Cre mice to L10-EGFP mice^34^, which express GFP in a Cre-dependent manner (hereafter referred to as Syn1-GFP mice). We observed expression of GFP in most neurons in the brain (including cerebrum, thalamus, midbrain, and brainstem) and a subpopulation of cells in the cerebellum in Syn1-GFP mice (Supplementary Fig. 3a–b). We did not observe clear differences in GFP expression between female and male Syn1-GFP mice (Supplementary Fig. 3a). We also tested whether there were sex differences in recombination efficiency in neuronal Ca_V_1.2 cKO mice by Western blot. We probed cerebrum and cerebellum samples from male and female neuronal Ca_V_1.2 cKO and CTRL mice for Ca_V_1.2 protein. We observed similar basal levels of Ca_V_1.2 expression in both sexes of CTRL mice, as well as similar degrees of Ca_V_1.2 deletion between female and male cKO mice (Supplementary Fig. 3c). We also observed that the cerebellum retains most Ca_V_1.2 expression in both female and male neuronal cKO mice (Supplementary Fig. 3c). We conclude that Syn1-Cre deletes Ca_V_1.2 from the majority of neurons in the brain and that sex differences in the behaviors of neuronal Ca_V_1.2 cKO mice are not likely to be driven by sex differences in Syn1-Cre-mediated Ca_V_1.2 deletion.

## Discussion

Ca_V_1.2 has important roles in neuronal function and has been linked to multiple neuropsychiatric disorders. Our behavioral data indicate that expression of Ca_V_1.2 in neurons is necessary for normal cognitive, social, and motor behavior. Deletion of Ca_V_1.2 from neurons resulted in gait differences and impaired associative learning in both sexes, as well as sex-specific alterations in motor learning, acoustic startle response, and social behaviors. Neuronal Ca_V_1.2 cKO mice used in this study have normal locomotor function and anxiety-like behavior, intact sensorimotor gating, and no signs of anhedonia.

Many studies have investigated the role of Ca_V_1.2 in behavior. Ca_V_1.2 haploinsufficient mice have a reduction in Ca_V_1.2 expression in all cell types throughout the body but some Ca_V_1.2 is still present to perform its normal functions (global Ca_V_1.2 deletion is lethal due to the dependence of the cardiovascular system on Ca_V_1.2 for development and function^39^). In contrast, deletion mediated by the Syn1-Cre transgene (this study) results in total loss of Ca_V_1.2 expression specifically in neurons^31^. Some studies have also used Nestin-Cre to delete Ca_V_1.2, which results in loss of Ca_V_1.2 in central and peripheral system neurons as well as glia. Yet other studies have used Cre driver lines and viral Cre expression approaches which target a more limited number of neurons or a more focal region of the brain than the approach we used. We will compare our results to those described in Ca_V_1.2 haploinsufficient mice and mice in which Ca_V_1.2 has been deleted using other Cre-based approaches.

Neuronal Ca_V_1.2 cKO mice used in this study exhibit normal locomotor activity, abnormal gait, and sex-specific alterations in motor learning. Previous work in Ca_V_1.2 haploinsufficient mice found no differences in motor learning but did find a hypoactive phenotype^11^. However, work in a more specific forebrain glutamatergic neuronal Ca_V_1.2 deletion (via CaMKII-Cre) found no changes in swim speed^32^ which concurs with our data showing that neuronal Ca_V_1.2 deletion does not induce baseline locomotor deficits. Interestingly, an analysis of gait and paw positioning in male Ca_V_1.2 haploinsufficient mice observed reduced stride duration at P30 which normalized by P60^15^. This contrasts with our data from adult mice showing increased step time in neuronal Ca_V_1.2 cKO mice during Erasmus Ladder. Increased step time in our neuronal Ca_V_1.2 cKO mice may contribute to the reduced number of missteps we observed on day 1 of the Erasmus Ladder compared to CTRL littermates, although all groups improved on this measure over days 2–4 and ultimately were not statistically different by day 4. Overall, we conclude that neuronal Ca_V_1.2 expression is important in specific components of motor learning and gait.

We observed an associative learning deficit on the Erasmus Ladder in our neuronal Ca_V_1.2 cKO mice that generalized across sexes. Ca_V_1.2 has previously been associated with a variety of learning and cognitive behaviors. Intriguingly, mice where Ca_V_1.2 is deleted in neurons and glia (via Nestin-Cre) display a learning deficit in operant conditioning that appears to be secondary to using a different learning strategy than CTRL mice^40^. It would be interesting to study whether our neuronal Ca_V_1.2 cKO mice learn the Erasmus Ladder cues less quickly than CTRL mice or use an entirely different decision-making strategy that results in the appearance of impaired learning. Although we did not perform Morris water maze, Temme et al. performed an interesting variation of this task on the same neuronal Ca_V_1.2 cKO mice, which showed that they perform normally on the standard Morris water maze but their performance weakens compared to CTRL littermates when cues are limited^29^. If Ca_V_1.2 is deleted in specific neuronal subtypes such as glutamatergic forebrain neurons (via CaMKII-Cre) or D1 receptor-expressing neurons (via D1-Cre), these cKO mice show normal learning in the Morris water maze at initial training but deficits appear if they are tested 30 days later^14,32^. Taken together, these studies show that Ca_V_1.2 is important in a variety of associative learning paradigms.

Previous work in mice with altered Ca_V_1.2 expression has identified sex-specific phenotypes. We found that male neuronal Ca_V_1.2 cKO mice did not display a preference for social interaction over object exploration to a statistically significant degree, whereas female neuronal Ca_V_1.2 cKO mice preferred social interaction over objects but overall engaged less in social activity than female CTRL littermates. While male Ca_V_1.2 haploinsufficient mice appear to display normal social preference^15^, mice with a more specific forebrain glutamatergic neuronal Ca_V_1.2 deletion (via CaMKII-Cre) exhibit impaired social preference^41^. This latter study^41^ did not specify sex and used a different genetic background than our work, but these results imply that the social preference phenotype might be driven by forebrain glutamatergic neurons. Our sex-specific results are especially interesting in the context of autism spectrum disorder which is both more common and manifests differently in males than females, and has been linked genetically with *CACNA1C*^9,23–26^. Overall, our data support the conclusion that there is a sex-specific effect of neuronal loss of Ca_V_1.2 in mice such that females have reduced social interaction overall while retaining social preference, and males do not display social preference.

Other sex-specific effects have been noted previously in the acoustic startle response. We found that neuronal Ca_V_1.2 cKO males had a decreased acoustic startle response and there were no deficits in neuronal Ca_V_1.2 cKO females. Another group previously reported the opposite finding in Ca_V_1.2 haploinsufficient mice: male mice had no deficits, yet haploinsufficient female mice had a decreased acoustic startle response^11^. Our finding of male-specific diminished startle response with intact prepulse inhibition is a notable finding, given that in human patients, lesions of the orbitofrontal cortex and amygdala can reduce baseline startle amplitude^42–46^. Diminished startle responses have also been observed in patients with disorders that have been genetically linked to Ca_V_1.2, including bipolar disorder^47^ and schizophrenia^48,49^, both of which have some evidence for differences in clinical manifestation between male and female patients^50–52^. More work will be required to understand the mechanism underpinning the role of Ca_V_1.2 in startle responses.

Neuronal Ca_V_1.2 cKO mice used in this study have normal anxiety-like behavior, which was also observed by a different group in this same neuronal cKO model^28^. However, several other Ca_V_1.2-deficient mice display abnormal anxiety phenotypes including haploinsufficient females^11,12^, CaMKII-Cre^12^, D1-Cre^14^, and AAV knockdown targeting the prefrontal cortex and nucleus accumbens^12,41,53^. One possible explanation for this discrepancy is that our conditional deletion strategy targets both excitatory and inhibitory neurons (among other types) and this may cause pleiotropic effects that obscure the contribution of excitatory neuron Ca_V_1.2 to anxiety behaviors. Further work would be required to explore this possibility. It is also possible that another Ca_V_1 channel may compensate for the loss of Ca_V_1.2; Ca_V_1.2 is upregulated when Ca_V_1.3 is deleted from pancreatic β cells^54^ and cardiomyocytes^55^, although the reciprocal (Ca_V_1.3 compensating for Ca_V_1.2) has not been reported in the literature. Such compensation may contribute to the differences in behavioral phenotypes noted when comparing neuronal Ca_V_1.2 knockouts and Ca_V_1.2 haploinsufficient mice in general. Furthermore, Ca_V_1 channels have well-defined presynaptic and postsynaptic functions in neurons^1–4^, but recent work has revealed a crucial role for Ca_V_1.2 in regulating myelination and proliferation of oligodendrocytes^56–58^. Anxiety-like phenotypes and abnormal fear learning are observed in mice where Ca_V_1.2 is deleted in glia as well as neurons^11,12,14,59^ and in some neuron-specific conditional cKO mouse models^12,14,41,53^, but not in Syn1-Cre neuron-specific conditional knockouts^28,29^ including the work reported here. Strain differences may also contribute to these discrepancies, as most of the published studies cited use C57BL/6J mice whereas we use a C57BL/6N × 129SvEv F2 hybrid line. Finally, not all cerebellar neurons are targeted by Syn1-Cre, and therefore there may be roles for Ca_V_1.2 in cerebellar neurons that are not identified in our work. Further work with mouse lines designed to specifically target glial populations, as well as lines that target neurons in spatially and temporally specific ways will be necessary to fully understand the role of Ca_V_1.2 in the brain.

## Summary

In summary, our data demonstrate that neuronal Ca_V_1.2 plays sex-specific roles in motor learning, acoustic startle response, and social preference, while effects on gait and associative learning generalize across sexes. In contrast to some studies, we found no evidence of hypoactivity or anxiolytic behaviors in neuronal Ca_V_1.2 cKO mice. This suggests that perhaps Ca_V_1.2 function in certain specific neuronal populations may be compensated for by other neurons in circuits involved in anxiety. This work contributes to our understanding of the complex role that Ca_V_1.2 may play in the motor, cognitive, social, and affective abnormalities in human neuropsychiatric disorders.

## Supplementary Information

Supplementary Information 1.

Supplementary Information 2.

Supplementary Information 3.

## Data Availability

The datasets generated during this study are available from the corresponding author on reasonable request.
