# Supplementary material for: Neuronal deletion of CaV1.2 is associated with sex-specific behavioral phenotypes in mice
Source: Sci Rep. 2022 Dec 22;12:22152. doi: 10.1038/s41598-022-26504-4 (PMC9780340; doi:10.1038/s41598-022-26504-4)
Supplement: Supplementary file 1 — Supplementary Information 1. [file 41598_2022_26504_MOESM1_ESM.pdf]

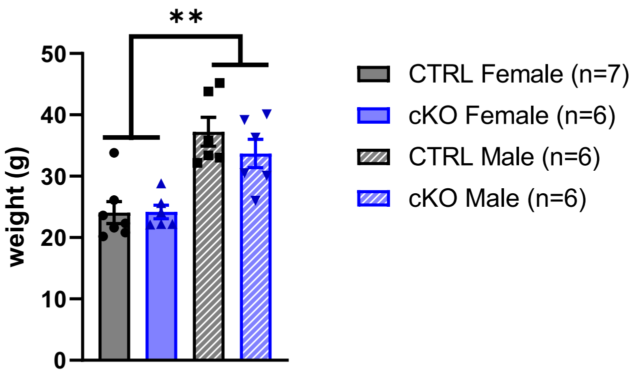

Supplementary Fig. 1

Neuronal $Ca_V1.2$ cKO mice had no differences in weight compared to wildtype littermates. **a** Female mice were significantly smaller compared to males. Weight, age 10-18 weeks at time of weight. (n = 6-7 per group). Data are expressed as mean ± s.e.m.
