# Supplementary material for: Neuronal deletion of CaV1.2 is associated with sex-specific behavioral phenotypes in mice
Source: Sci Rep. 2022 Dec 22;12:22152. doi: 10.1038/s41598-022-26504-4 (PMC9780340; doi:10.1038/s41598-022-26504-4)
Supplement: Supplementary file 2 — Supplementary Information 2. [file 41598_2022_26504_MOESM2_ESM.pdf]

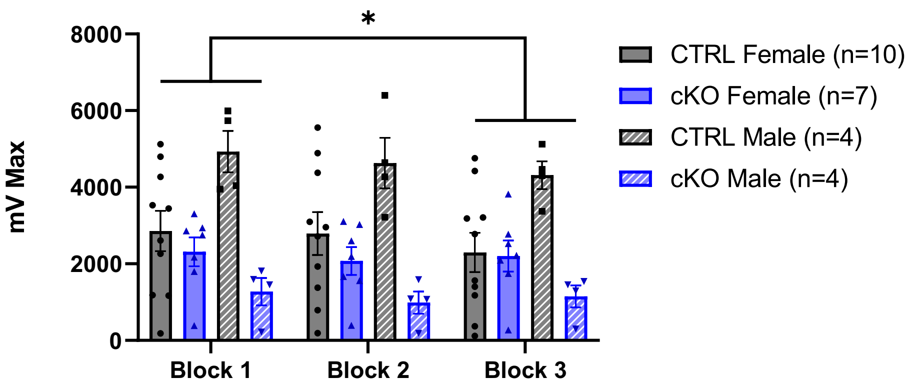

Supplementary Fig. 2

There was a decrease in startle response for all groups over the course of the experiment. Acoustic startle response, habituation. (n = 4-10 per group). Data are expressed as mean ± s.e.m.
