# Supplementary material for: Neuronal deletion of CaV1.2 is associated with sex-specific behavioral phenotypes in mice
Source: Sci Rep. 2022 Dec 22;12:22152. doi: 10.1038/s41598-022-26504-4 (PMC9780340; doi:10.1038/s41598-022-26504-4)
Supplement: Supplementary file 3 — Supplementary Information 3. [file 41598_2022_26504_MOESM3_ESM.pdf]

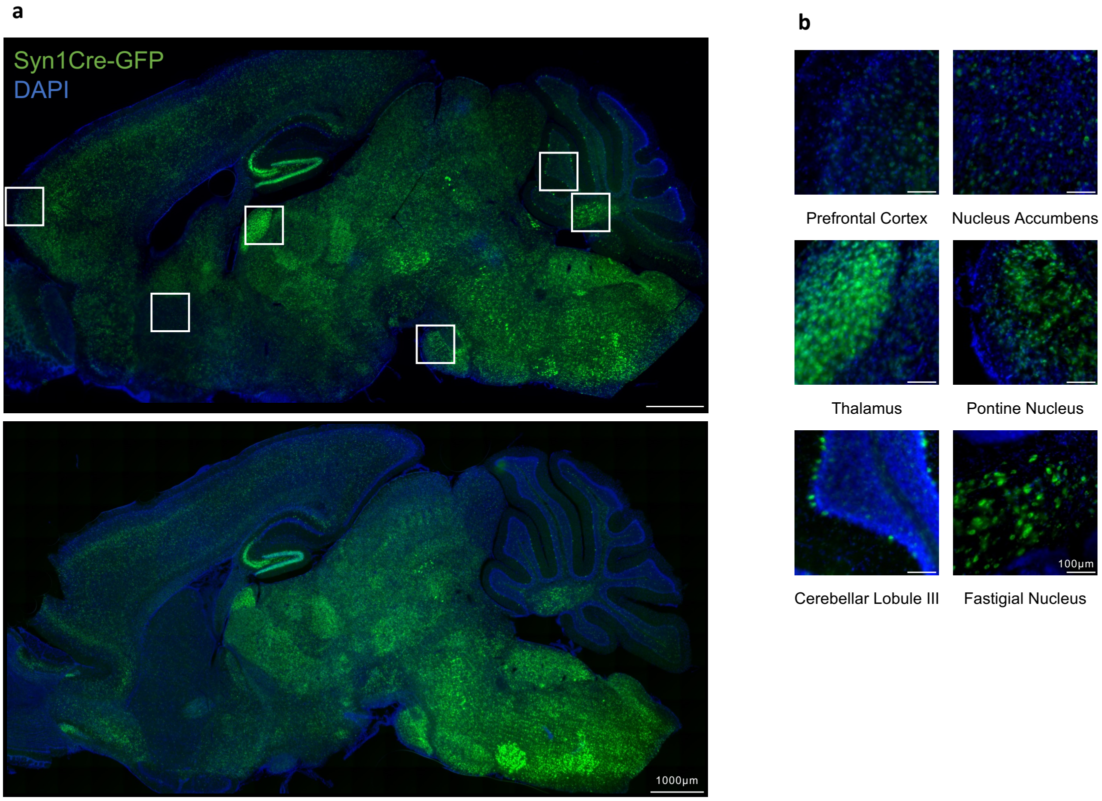

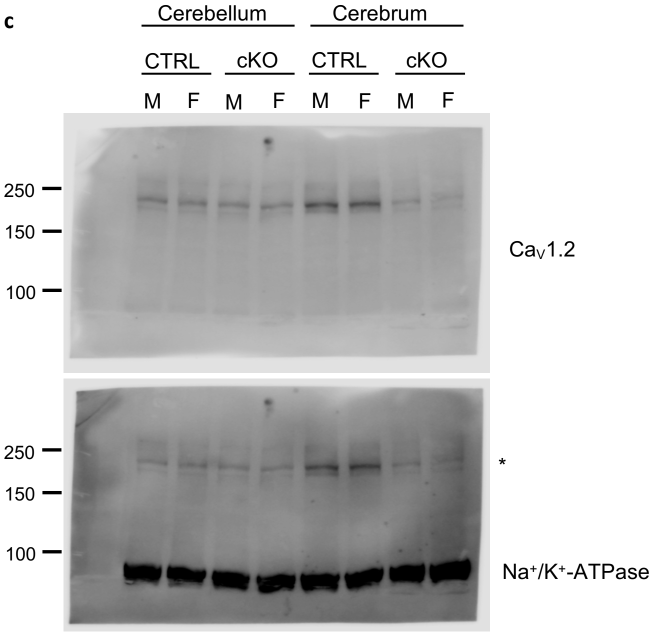

Supplementary Fig. 3

Synapsin1-Cre mediates similar patterns and levels of recombination between sexes. **a** Female (top) and male Syn1-GFP (bottom) mice showing broad expression of GFP across most brain regions with largely similar expression patterns between sexes, using Olympus cellSens Dimension 2.3, www.olympus-lifescience.com/en/software/cellsens. **b** Higher magnification images of GFP in Syn1-GFP mice in regions indicated by squares in **a**. **c** Western blot shows that the cerebellum retains most $Ca_V1.2$ expression in neuronal $Ca_V1.2$ cKO mice whereas the cerebrum loses most $Ca_V1.2$ expression (upper blot). $Ca_V1.2$ protein expression is decreased to a similar degree in male (M) and female (F) neuronal $Ca_V1.2$ cKO mice. Na^+/K^+-ATPase is shown as a loading control (lower blot). Asterisk (*) in lower blot indicates residual $Ca_V1.2$ antibody.
